# NeuroimaGene: an R package for assessing the neurological correlates of genetically regulated gene expression

**DOI:** 10.1186/s12859-024-05936-x

**Published:** 2024-10-08

**Authors:** Xavier Bledsoe, Eric R. Gamazon

**Affiliations:** 1 Medical Scientist Training Program, Vanderbilt University, Nashville, TN USA; 2 Vanderbilt Genetics Institute, Vanderbilt University Medical Center, Nashville, TN USA; 3 Vanderbilt Memory & Alzheimer’s Center, Nashville, TN USA

**Keywords:** Transcriptomics, Neuroimaging, Psychiatry, Neurology, Genomics

## Abstract

**Background:**

We present the NeuroimaGene resource as an R package designed to assist researchers in identifying genes and neurologic features relevant to psychiatric and neurological health. While recent studies have identified hundreds of genes as potential components of pathophysiology in neurologic and psychiatric disease, interpreting the physiological consequences of this variation is challenging. The integration of neuroimaging data with molecular findings is a step toward addressing this challenge. In addition to sharing associations with both molecular variation and clinical phenotypes, neuroimaging features are intrinsically informative of cognitive processes. NeuroimaGene provides a tool to understand how disease-associated genes relate to the intermediate structure of the brain.

**Results:**

We created NeuroimaGene, a user-friendly, open access R package now available for public use. Its primary function is to identify neuroimaging derived brain features that are impacted by genetically regulated expression of user-provided genes or gene sets. This resource can be used to (1) characterize individual genes or gene sets as relevant to the structure and function of the brain, (2) identify the region(s) of the brain or body in which expression of target gene(s) is neurologically relevant, (3) impute the brain features most impacted by user-defined gene sets such as those produced by cohort level gene association studies, and (4) generate publication level, modifiable visual plots of significant findings. We demonstrate the utility of the resource by identifying neurologic correlates of stroke-associated genes derived from pre-existing analyses.

**Conclusions:**

Integrating neurologic data as an intermediate phenotype in the pathway from genes to brain-based diagnostic phenotypes increases the interpretability of molecular studies and enriches our understanding of disease pathophysiology. The NeuroimaGene R package is designed to assist in this process and is publicly available for use.

**Supplementary Information:**

The online version contains supplementary material available at 10.1186/s12859-024-05936-x.

## Background

The NeuroimaGene resource seeks to identify the aspects of brain structure and function that are affected by the expression of individual genes or gene sets. Transcriptome-wide association studies (TWAS) of brain-related phenotypes have implicated genetically regulated expression (GReX) for hundreds of genes as potential components of disease pathophysiology [1]. By focusing on transcriptional regulation, TWAS methodologies dramatically improve the interpretability of GWAS studies [2]. Nevertheless, understanding the consequences of GReX remains difficult, especially in brain-based conditions. The pathways linking GReX through molecular, cellular, and organ-level architecture all the way to symptomatology are challenging to characterize.

**Fig. 1:**
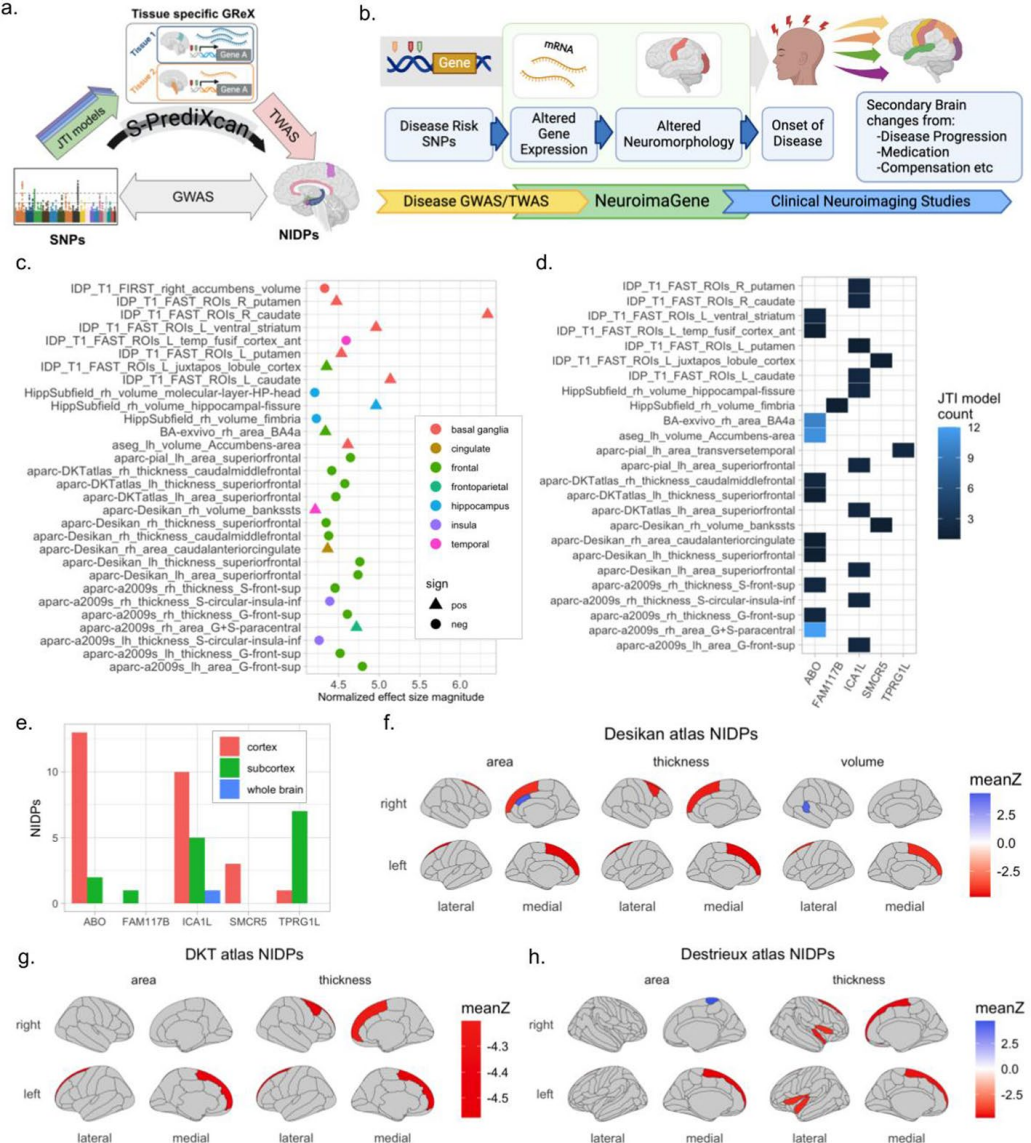
Overview of NeuroimaGene pipeline and data from worked example. **a** Schematic of the TWAS methodology. **b** Visual representation of the NeuroimaGene resource in context linking genes with neuroimaging features in a hypothetical disease-associated pathway. **c** Distribution of aggregate effect sizes of stroke-associated genes on all associated NIDPs. **d** Heatmap illustrating the significant associations between GReX (x-axis) and NIDPs (y-axis) with colored tiles representing the number of tissue contexts in which the association met statistical significance. **e** Distribution of significant associations between NIDPs (y-axis) and stroke-associated genes (x-axis) with NIDPs grouped according to the type of brain region they characterize. **f**–**h** 2 dimensional representations of affected brain regions according to the Desikan atlas (**f**), the DKT atlas (**g**), and the Destrieux atlas (**h**). Colors represent the mean normalized effect size across all gene-level associations for each region

An effort to understand the contribution of gene-level associations to neuropsychiatric traits would benefit from the presence of a quantitative endophenotype that is both associated with molecular findings as well as the symptomatic experiences of individuals (Fig. 1a). We select magnetic resonance imaging (MRI) of the brain as this endophenotype. Clinical studies continue to identify neuroimaging profiles associated with a wide variety of conditions [3–5]. Additionally, many neuroimaging measures themselves demonstrate significant SNP-driven heritability [6].

We recently performed transcriptome wide association studies for over 3500 neuroimaging derived phenotypes (NIDPs) captured in the UK biobank [7]. These NIDPs span different MRI modalities including T1, dMRI, and fMRI which characterize regional brain structure, white matter characteristics, and functional coactivation, respectively. We applied the S-PrediXcan methodology to GWAS summary statistics for each NIDP (Fig. 1a). These summary statistics are hosted on the Oxford Brain Imaging Genetics Server, version BIG40 26/03/21. For gene expression models, we used joint tissue imputation enriched models derived from GTEx v8 data and described in Zhou et al. [8]. To optimize for neurological interpretability, we rely on the 13 models trained on brain regions as well as 6 tissues reflecting the neurologically relevant systems. These include the immune system (whole blood), blood toxicity (liver), the gut-brain axis (sigmoid and transverse colon models), and the endocrine feedback system (pituitary and adrenal gland). These models are publicly accessible on Zenodo at 10.5281/zenodo.3842289 (version 1).

Following extensive quality analyses and filtering, we packaged these tissue-specific GReX-NIDP associations into a resource. This resource, called NeuroimaGene, represents a searchable repository of associations between GReX in 19 different tissue contexts and 3547 NIDPs. Pre-existing resources such as the Allen Brain atlas and FUMA (Functional Mapping and Annotation of Genome-Wide Association Studies) classify the spatial expression of genes in the human brain [9, 10]. These resources describe the experimentally assayed expression of RNA transcripts across different aspects of the brain. These resources do not describe where the effects of local gene expression changes manifest. The interconnected nature of the brain with long ranging axonal connections permits non-local effects of gene expression. NeuroimaGene extends the functionality of these pre-existing resources to quantitatively identify the effect of GReX in specific brain regions on brain features both local and distal to the region of implicated gene expression.

We have packaged the NeuroimaGene resource into a user-friendly, open access R package for public use. The primary function of the NeuroimaGene R package is to identify neuroimaging derived brain features that are impacted by the GReX of user-provided genes or gene sets. This resource can be used to (1) characterize individual genes or gene sets as relevant to the structure and function of the brain, (2) identify the region(s) of the brain or body in which expression of target gene(s) is neurologically relevant, (3) impute the brain features most impacted by user-defined gene sets such as those produced by genome-wide and transcriptome wide association studies, and (4) generate publication level, modifiable visual plots of NeuroimaGene associations.

## Implementation

### Installation

NeuroimaGene is an open source R package that can be downloaded from the comprehensive R archive network (CRAN) using R’s built in “install.packages()” function (https://doi.org/10.32614/CRAN.package.neuroimaGene) The repository for the R package is located on GitHub (https://github.com/xbledsoe/NeuroimaGene_R). NeuroimaGene requires no formal external dependencies other than R (≥ 3.5.0) and imports six well-documented packages on installation that are necessary for functionality (data.table, ggplot2, DBI, stringr, ggseg, and RSQLite). The R package, hosted on CRAN and GitHub, represents a set of functions and supporting files designed to interrogate the NeuroimaGene database stored on Zenodo. Upon attempting to use the neuroimaGene() command for the first time, the user will be prompted to download the database from its permanent location on Zenodo (https://doi.org/10.5281/zenodo.10994978). The current database may be expanded, as new data are compiled. A minimally sufficient subset of the database is included in the package which can be used to run the example scripts included in the official documentation.

### Usage

NeuroimaGene is designed to be accessible to users with minimal coding experience. A comprehensive manual in PDF format as well as a vignette with worked examples are accessible on CRAN. The package provides functionality for users to interrogate the local context in which target genes are neurologically relevant and to identify the primary brain regions impacted by the GReX of these genes.

### Querying associations between GReX and NIDPs

The primary NeuroimaGene query, ‘neuroimaGene()’, returns a set of tissue-specific associations between the GReX of user-defined genes and NIDPs of interest. This function takes a vector of either HUGO gene names or ENSEMBL gene IDs as necessary user input. Second, the user has three options available to restrict the NIDPs queried by the neuroimaGene command. The ‘modality’ parameter allows the user to restrict the query to NIDPs derived from T1 structural MRI, diffusion tensor imaging, or functional MRI. The ‘atlas’ parameter, allows the user to further restrict the query to named cortical atlases such as the Desikan-Killiany (DK) or Destrieux atlases when using T1 modalities. Within diffusion tensor imaging, there are two ‘atlas’ options which reflect different algorithms used to infer biology from the diffusion data. These are probabilistic tractography and tract based spatial statistics. A full list of modalities and atlases are available to browse in the help vignette and on the GitHub README. To use all modalities, or all atlases within a modality, the user must set the corresponding parameter to either NA or ‘all’. Third, the user can input a vector of pre-determined NIDPs. These NIDPs must match the names used in NeuroimaGene exactly. When neuroimaging nomenclature from outside imaging studies differs from the NeuroimaGene NIDP names, matching NIDPs must be identified manually. The ‘listNIDPs()’ function will return all NIDP names according to modality and atlas parameters that are identical to those in the neuroimaGene() function.

Lastly, the user will have the option to identify a multiple testing correction procedure for the statistical significance threshold. Each imaging modality or atlas contains a different number of NIDPs. The Bonferroni correction (‘BF’) treats each of these NIDPs as independent even though data analyses demonstrate that this is not accurate [7]. This is a highly conservative threshold that will yield high confidence associations but is likely to generate many false negatives. Nevertheless, users may select this threshold using the mtc = ‘BF” parameter. Recognizing the correlation of brain measures from the same modality and atlas, we set the default multiple testing correction to reflect the less stringent Benjamini Hochberg (‘BH’) false discovery rate. Users are also permitted to access all nominally significant results (< 0.05, uncorrected) by setting the multiple testing correction parameter to ‘nom’.

The multiple testing correction parameter represents a study-wide threshold and is therefore dynamic, depending on the modality and atlas parameters provided in the initial neuroimaGene query. If a user provides an atlas, multiple testing correction will be calculated for all tissues, NIDPs and genes for the NIDPs in that atlas. If the user provides a modality such as ‘T1’ of ‘dMRI’ but sets the atlas as NA or ‘all’, multiple testing correction will be calculated for all associations involving NIDPs from that modality. If the user sets the both the modality and atlas to NA or ‘all’, the correction will be applied to the entire data set of all 19 tissue models and 3537 NIDPs and 22,436 genes. This format applies to both the Bonferroni (BF) and Benjamini Hochberg (BH) corrections with an alpha of 0.05 in each case. Users wishing to provide their own vector of NIPDs will receive results according to a nominal threshold and will be alerted of the necessity to perform multiple testing correction themselves. When run, the neuroimaGene command returns a data.table object describing tissue-specific associations between gene expression and NIDPs. Descriptions of each data column in the neuroimaGene object are further provided in the package documentation.

### Performance benchmarking

The neuroimaGene() function is the primary function of the package in which user-defined gene sets are associated with NIDPs from the UKB. We perform benchmarking analyses for the performance of this function regarding runtime and memory allocation within R (Supplementary Figs. 1–2, Additional file 1). We perform 100 iterations of the neuroimaGene command on gene sets of multiple lengths across all 3 multiple testing thresholds and 5 different atlas/modality parameters. The most computationally demanding analysis completed neuroimaGene analysis of 150 genes using a nominal threshold. Across all 5 tested atlas and modality parameters, the maximal mean runtime was under 7 s. The memory allocated across our analyses scaled linearly with the number of genes assessed and achieved a maximum of 2 MB in our benchmarking analyses. The NeuroimaGene packages relies on an external SQL database (1.9 GB) for which automatic download permissions are requested immediately upon package installation.

### Visualization

Once a query has returned data in the form of a table, there are several options for visualizing the findings. NeuroimaGene implements customized functions based on the ggplot and ggseg packages to generate visual output [11, 12]. The plot_gns() function takes the results table as an input and returns a ggplot bar chart showing the number of NIDPs (y-axis) associated with each gene (x-axis). By default, the plot only displays the top 15 genes as ranked by top effect size. The maximum number of genes to be displayed can be modified using the maxGns parameter.

Complementary to the plot_gns() function is the plot_nidps() function. This command takes the neuroimaGene results table as input and returns a ggplot dot plot object showing the aggregate effect size magnitude of all significantly associated query genes on the NIDPs from the results table. Effect sizes are aggregated by simple arithmetic mean. The maxNIDPs parameter can be set manually to show more NIDPs than the default 30. By default, the function displays a comparison of the normalized effect size magnitude with direction of effect indicated by the point shape. The user can also specify that the function display the normalized effect size as a vector rather than a scalar by using the mag = FALSE parameter.

The plot_gnNIDP() function takes the results table as input and returns a ggplot heatmap object. The x-axis represents the queried genes, and the y-axis shows the associated NIDPs. The data in the plot represent associations between GReX of the x-axis genes and the y-axis NIDPs colored according to the number of tissue contexts in which the association was determined to be statistically significant. Default gene and NIDP counts can be adjusted using the previously described parameters.

Lastly, the neuro_vis() command leverages the ggseg and fsbrain packages to generate visual representations of cortical and subcortical GReX associations [12, 13]. This function takes as input a NeuroimaGene results data table. Notably, this script is limited to displaying visual representations of the DK, DKT, Destrieux, and subcortical segmentation atlases only. The appropriate atlas must be included with the atlas parameter. The function returns a two-dimensional multi-panel plot with NIDPs colored according to the aggregate effect size of all associated genes from the results table. The high, mid, and low-range color scales default to blue, white, and red, and can be customized via function parameters.

## Results

As an example of the utility of the NeuroimaGene package, we applied the functions to gene-level data from a transcriptome-wide association study of stroke (Fig. 1b). Mishra et al. identified 27 genes whose genetically regulated expression is associated with stroke in cross-ancestry GWAS meta-analyses of 110,182 individuals who have experienced strokes and 1.5 million controls [14]. We compiled the gene names for these 27 genes into a vector named ‘stroke_gns’ and executed the following command:

- ng <- neuroimaGene(stroke_gns, modality = 'T1', atlas = 'all', mtc = 'BH')

This produced a data.table object with associations between GReX for 5 genes and 43 NIDPs across 17 of the 19 available tissue contexts (Additional file 2). Using the plot_nidps(), plot_gnNIDP(), and plot_gns() functions, we can visualize these associations graphically (Fig. 1c-e). Each association between a stroke gene and an NIDP can be detected in any of the 19 different tissue models in the resource. Each tile in the heatmap (Fig. 1d) is thus colored according to the number of different tissue models in which the association passed the user-defined significance threshold. The strongest finding by effect size is *ICA1L*, which is strongly associated with the area of the left superior frontal gyrus. This association is detected in 4 different cortical atlases (pial, DKT, Desikan, and the a2009 Destrieux atlas). We visualize these findings using the mag = FALSE parameter as well (Supplementary Fig. 3, Additional file 1). Additionally, the NIDPs that showed the largest magnitude effect from the GReX of stroke-associated genes are the right and left caudate. This finding is also driven by *ICA1L.* Using the neuro_vis() function, we then generated visual depictions of the brain regions most impacted by the expression of the 5 significant stroke-associated genes in the Desikan, DKT, and Destrieux atlases (Fig. 1f-h).

## Discussion

Using the NeuroimaGene package enables quick and streamlined identification of brain features that are associated with the genetically regulated expression of genes identified from disease studies. In the example above, we identify 5 genes from a stroke GWAS whose expression is associated with changes in the morphology of brain regions in healthy patients. This suggests that the expression of these genes is relevant to neurologic health, even in the absence of stroke history. Of the 27 genes identified by Mishra et al. our data highlight *ICA1L* and *ABO* as being of particular interest owing to the broad range of associations with NIDPs. Our data suggest that the genes have a disproportionate effect on the caudate nuclei and the superior frontal gyri. Supporting the utility of NeuroimaGene as a gene prioritization schema, multiple protein-level analyses indicate that the ICA1L protein is associated with cerebral small vessel disease and small vessel stroke [15, 16]. All associations for *ICA1L* were detected in exactly 2 tissue contexts: the cortex and the frontal cortex. These findings suggest that not only is ICA1L expression associated with stroke risk, but that expression in the cortex is of particular importance. The *ABO* gene determines blood type which has been widely characterized as a risk factor for stroke [17]. While the top two genes represent known stroke risk factors, NeuroimaGene prioritizes three additional genes as being associated both with endogenous brain structure and stroke risk.

Here we have used a neurologic condition, stroke, as our phenotype of interest. In the case of psychiatric conditions, the NeuroimaGene resource provides an additional layer of experimental possibility. Neuroimaging studies of schizophrenia and anxiety disorders have already identified NIDPs (fMRI, dMRI, and T1 modalities) that are associated with the diagnosis [18–20]. Applying NeuroimaGene to the TWAS findings for these conditions will generate sets of predicted neurologic changes that are associated with GReX of the disease-associated genes. As we demonstrated in previous work, comparison of gene-derived neuroimaging features and empiric neuroimaging features permits the hypothetical annotation of clinical neuroimaging findings with specific disease-associated genes. This process is described in the context of schizophrenia in a prior publication [7]. It is crucial to ensure consistency between the NeuroimaGene atlas and the atlas used in the clinical study. Fortunately, the wide breadth of neuroimaging atlases used in NeuroimaGene favors compatibility with a wide array of neuroimaging studies. While NeuroimaGene is currently limited to associations derived from joint tissue imputation gene expression models and NIDPs derived from the UK biobank, the model of analysis we present here is publicly available and can be applied to single cell, or developmental models as well as neuroimaging data from other resources.

## Conclusions

NeuroimaGene is a user-friendly, open access R package now available for public use. The primary function of the tool is to identify neuroimaging derived brain features that are impacted by the GReX of user-provided genes or gene sets. This resource can be used to (1) characterize individual genes or gene sets as relevant to the structure and function of the brain, (2) identify the region(s) of the brain or body in which expression of target gene(s) is neurologically relevant, (3) impute the brain features most impacted by user-defined gene sets such as those produced by GWAS and TWAS, and (4) generate publication level, modifiable visual plots of NeuroimaGene associations.

### Availability and requirements

- Project Name: neuroimaGene
- Project Home Page: https://github.com/xbledsoe/NeuroimaGene_R
- Operating System: Platform independent
- Programming Language: R
- Other Requirements: R (≥ 3.5.0)
- License: GNU GPL
- Any restrictions to use by non-academics: none

## Supplementary Information

Additional file 1

Additional file 2

## Data Availability

The R package for the current study is available at https://github.com/xbledsoe/NeuroimaGene_R and on CRAN at 10.32614/CRAN.package.neuroimaGene. The NeuroimaGene SQL database is hosted permanently on Zenodo at 10.5281/zenodo.10994978.

## Abbreviations

GReX: Genetically regulated gene expression
NIDP: Neuroimaging derived phenotype
GWAS: Genome wide association study
TWAS: Transcriptome wide association study
MRI: Magnetic resonance imaging
dMRI: Diffusion MRI
fMRI: Functional MRI
