# Supplementary material for: NeuroimaGene: an R package for assessing the neurological correlates of genetically regulated gene expression
Source: BMC Bioinformatics. 2024 Oct 8;25:325. doi: 10.1186/s12859-024-05936-x (PMC11463069; doi:10.1186/s12859-024-05936-x)
Supplement: Supplementary file 1 — Additional file 1 [file 12859_2024_5936_MOESM1_ESM.docx]

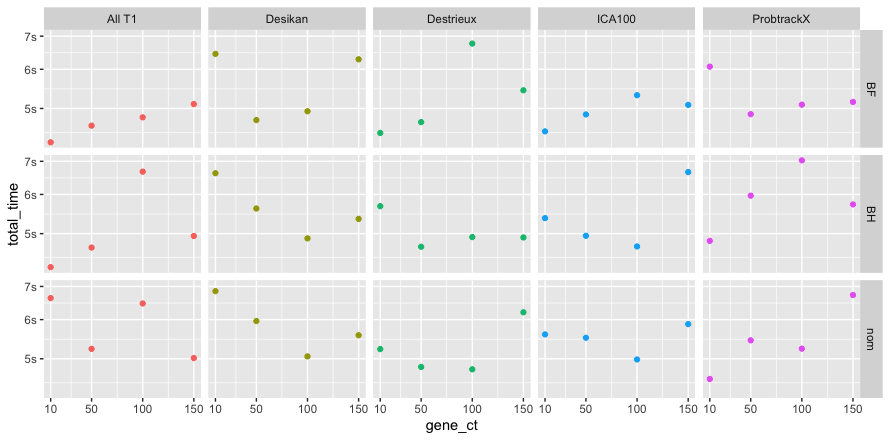


Supplementary Figure 1: Benchmarking of the NeuroimaGene() function according to total run time. The total runtime is listed in seconds on the y axis. The number of genes assessed by NeuroimaGene in each benchmarking test are listed on the x axis. Panels are faceted according to select atlas parameters as columns and the three multiple testing parameters as rows.


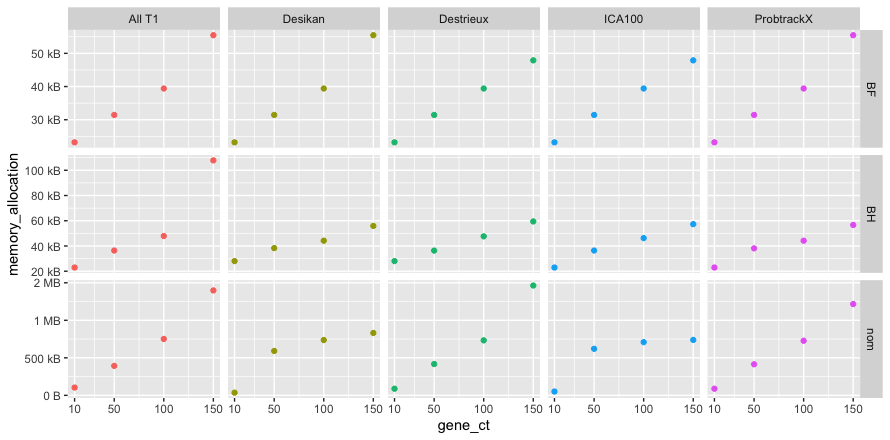


Supplementary Figure 2: Benchmarking of the NeuroimaGene() function according to total memory allocation. The total memory allocation is listed on the y axis. The number of genes assessed by NeuroimaGene in each benchmarking test are listed on the x axis. Panels are faceted according to select atlas parameters as columns and the three multiple testing parameters as rows.

**
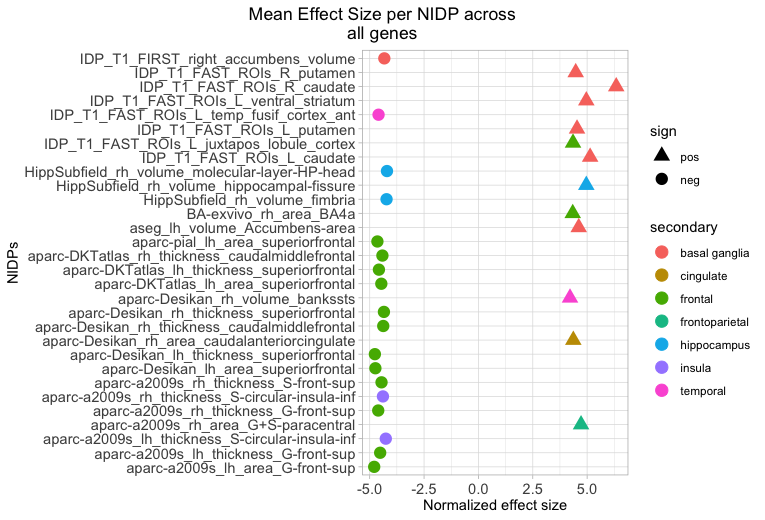
**

Supplementary Figure 3: Distribution of aggregate effect sizes of stroke-associated genes on all associated NIDPs. Points are colored according to the brain regions in which they lie and the shape represents the sign of the mean effect size of all significantly associated stroke genes.
